# Visual outcome following bilateral non-arteritic anterior ischemic optic neuropathy: a systematic review and meta-analysis

**DOI:** 10.1186/s12886-017-0543-y

**Published:** 2017-08-24

**Authors:** Shay Keren, Mario Zanolli, Gad Dotan

**Affiliations:** 1 Department of Ophthalmology, Tel Aviv Sourasky Medical Center, Tel Aviv, Israel; 2 Sackler School of Medicine, Tel Aviv University, Tel Aviv, Israel; 3 Department of Ophthalmology, Facultad de Medicina, Clinica Alemana de Santiago, Universidad del Desarrollo, Santiago, Chile; 4 Ophthalmology Department, Schneider Children’s Medical Center of Israel, Petach Tikva, Israel; 5 Ophthalmology Clinic, Schneider’s Children’s Medical Center of Israel, 14 Kaplan Street, PO Box 559, 49202 Petach Tikvah, Israel

**Keywords:** Non-Arteritic anterior ischemic optic neuropathy (NAION), Meta-analysis

## Abstract

**Background:**

Many patients who suffer unilateral non-arteritic anterior ischemic optic neuropathy (NAION) will eventually develop the same condition in their other eye, worrying them about losing vision in both eyes. The purpose of this meta-analysis is to determine whether it is possible to predict the visual outcome of the consecutive NAION event based on initial presentation and to compare mean visual loss of firstly versus secondly affected eyes.

**Methods:**

A systematic review and meta-analysis of studies published between January 1st 1966 and May 31st 2016 reporting on visual acuity and/or visual field loss of both affected eyes, measured either at presentation or follow-up following bilateral NAION.

**Results:**

Ten studies were included in the meta- analysis of visual acuity, including 9 retrospective reports and one randomized clinical trial, and five retrospective studies were included in visual field meta-analysis. A significant correlation exists for visual acuity (*R* = 0.387, *P* < 0.001) in both eyes of the same patient following bilateral NAION, and also for visual field loss (*R* = 0.445, *P* < 0.001) in the two eyes. The calculated coefficient of determination (R^2^) of 0.149 for visual acuity, and 0.198 for visual field loss indicates that for any given individual suffering from unilateral NAION only 15% of visual acuity and 20% of visual field loss in the secondly affected eye can be explained by these outcomes in the first eye. In addition, there was no difference in mean visual outcome of the first versus second NAION events (standardized mean differences of visual acuity 0.008, *P* = 0.890; and visual field loss, −0.019, *P* = 0.819).

**Conclusion:**

Even though a weak connection exists between visual outcome in both eyes following bilateral NAION it is still impossible to predict with certainty the visual outcome of a sequential contralateral NAION event based on the severity of visual loss in the first affected eye. Measures often taken after the first event are ineffective in improving the visual outcome of a second event should it occur.

## Background

Non-arteritic anterior ischemic optic neuropathy (NAION) is a potential cause of irreversible vision loss, typically occurring in patients over the age of 50 years [1]. Usually, at presentation one is affected; however, subsequent development of the same condition in the other eye is not uncommon; [2] occurring in approximately 15% of patients within a 5-year period [3]. Presumably, having similar optic disk anatomy in both eyes and exposure to the same vasculopathic risk factors may result in bilateral involvement of both optic nerves [4, 5]. Diabetics and patients who suffered significant visual loss following the first event are at increased risk for bilateral sequential involvement [3]. Unfortunately, no effective therapy is currently available that can prevent this occurrence. Thus, patients suffering from unilateral NAION are naturally concerned about the imminent possibility of losing vision in their other eye, and are often interested to know if it is possible to predict the visual outcome following the second event, should it occur, based on the initial presentation. Previous studies assessing visual outcome following bilateral NAION reported conflicting conclusions. Some authors found similar vision in affected eyes of the same individual, whereas others reported the opposite. This controversy remains unresolved even today.

In this study, we performed a systematic review and meta-analysis of studies reporting on visual outcome following bilateral NAION in attempt to resolve this conflict. In addition, we analyzed the severity of the first versus the second NAION event.

## Methods

This study was performed in accordance to a predefined protocol adhering to the guidelines of the Preferred Reporting Items for Systematic Reviews and Meta-Analyses (PRISMA) and the Mata-Analysis of Observational Studies in Epidemiology (MOOSE).

### Eligibility criteria

For this systematic review and meta-analysis, we considered population-based prospective and retrospective studies describing visual acuity and/or visual field assessments following bilateral NAION.

### Study selection

The analysis covered publications of adult patients (>18 years old) with bilateral NAION that included data of visual acuity and/or visual field loss of both affected eyes, measured either at presentation or follow-up. Publications were considered suitable for inclusion if they included raw data or statistical analysis. In case of multiple studies possibly reporting on the same population of patients (by the same authors) only the one reporting on the largest cohort of patients was included. Unpublished papers, nonhuman studies, letters to the editor, editorials, reviews, single case reports, studies enrolling children (<18 years old), and studies with no visual data were excluded from analysis. Studies reporting on bilateral optic neuropathy following Amiodarone use or studies in which it was impossible to ascertain NAION as the cause of visual loss were excluded as well.

### Information sources

A systematic literature search was performed by all authors on the PubMed, Ovid, and Google Scholar databases using the medical search headings and open text fields for publications from the last 50 years (January 1st 1966 to May 31st 2016). The reference list of retrieved articles was also searched for suitable papers.

The search terms included: “non-arteritic ischemic optic neuropathy” AND any of the following terms: “sequential”, “subsequent”, “successive”, “consecutive”, “second eye”, “fellow eye”, “bilateral” and “bilaterality”. Search criteria were translated according to the language of the database. A total of 112 publications were obtained. Duplicates were excluded by auto- and hand- searching [6]. All authors independently screened all the retrieved articles for inclusion and exclusion, and any disagreements were discussed and resolved. The Review Manager (RevMan) Computer program (Version 5.2. Copenhagen: The Nordic Cochrane Centre, The Cochrane Collaboration, 2012) was used for management of identified records.

### Data extraction and risk of bias assessment

Data collected from each study included: Study title, journal’s name, publication year, first author’s name, study design, and sample size. Visual acuity was recorded using the logarithm of minimal angle of resolution (Log MAR). Visual field loss was determined by automated perimetry, Goldmann perimetry, or tangent perimetry as long as the same technique was used for all follow-up evaluations, allowing correlation analysis between the two eyes. The correlation coefficient and/or the mean difference of the first and second NAION eyes were collected from studies conducting a statistical analysis. These statistical parameters were also calculated from studies reporting only raw data.

The potential for publication bias of studies reporting on visual outcome following bilateral NIAON was considered to be low because there is no benefit for either finding a strong or poor correlation of visual outcome between the two involved eyes.

### Statistical analyses

Meta-analysis was performed using the comprehensive meta-analysis software version 2 (Biostat Inc., Englewood, NJ). All tests were two-tailed and statistical significance was defined at an alpha level below 5%. A separate meta-analysis was conducted for visual acuity and visual field loss. The effect sizes calculated were Pearson’s correlation coefficient and standardized mean difference.

Studies heterogeneity was assessed using the I^2^ and Q tests. When I^2^ was greater than 50%, and Q was statistically significant, indicating significant heterogeneity between studies the random effect model was used for calculations. Otherwise the fixed effect model was used.

## Results

### Literature search

**Fig. 1:**
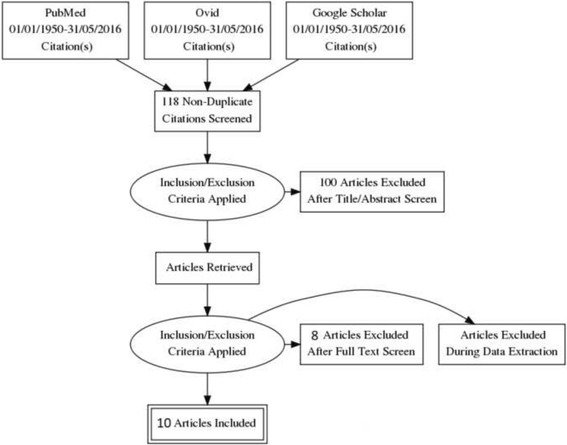
Flowchart of studies search, selection and inclusion for meta-analysis

The literature search and the manual search of references resulted in 118 papers evaluated for meeting inclusion criteria. Following exclusion 10 studies were included in analysis, including 9 retrospective reports and one prospective, randomized clinical trial (Fig. 1). Most publications were from the United States (*n* = 8), and the remaining two were from Israel, and Greece.

### Correlation of visual outcome in both eyes

**Fig. 2:**
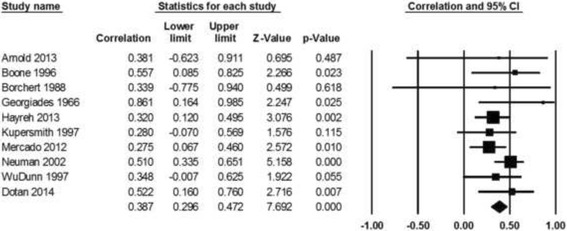
Correlation meta-analysis of visual acuity in both affected eyes of the same individual following bilateral NAION

**Fig. 3:**
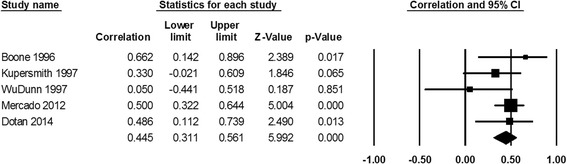
Correlation meta-analysis of visual field loss in both affected eyes of the same individual following bilateral NAION

The correlation coefficient of visual acuity between both eyes of the same patient following bilateral NAION calculated from data provided in 10 studies (Table 1) is statistically significant (*R* = 0.387, *P* < 0.001, Fig. 2). Based on data retrieved from 5 studies (Table 2), the correlation coefficient of visual field loss in both eyes after bilateral NAION is also statistically significant (*R* = 0.445, *P* < 0.001, Fig. 3).

**Table 1:** Studies included in the meta-analysis of visual acuity following bilateral NAION

Author	Year	Publication type	No. of patients	Correlation coefficient	Statistical Analysis (P or CI)
Studies that found correlation in visual outcome					
Boone et al. [11]	1996	Retrospective	23	0.522	0.043
Mercado et al. [12]	2012	Retrospective	86	0.276	0.01
Newman et al. [3]	2002	Prospective	48	0.36	CI 0.08-0.58
Studies that found no correlation in visual outcome					
WuDunn et al. [8]	1997	Retrospective	31	0.19	N/A
Kupersmith et al. [10]	1997	Retrospective	33	0.28	CI −0.07-0.57
Georgiades et al. [13]	1966	Retrospective	17	0.26	
Hayreh et al. [9]	2013	Retrospective	174	0.33	CL 0.24-0.40
Dotan et al. [7]	2014	Retrospective	25	0.279	0.176
Studies with only raw data					
Arnold et al. [1]	2013	Retrospective	108	0.381	0.227
Borchert et al. [14]	1988	Retrospective	10	0.547	0.339

**Table 2:** Studies included in the meta-analysis of visual field loss following bilateral NAION

Author	Year	Publication type	No. of patients	VF correlation	Sig. (P or CI)
Studies that found correlation in visual outcome					
Boone et al. [11]	1996	Retrospective	23	0.622	0.039
Mercado et al. [12]	2012	Retrospective	86	0.50	<0.001
Studies that found no correlation in visual outcome					
WuDunn et al. [8]	1997	Retrospective	31	−0.05	N/A
Kupersmith et al. [10]	1997	Retrospective	33	0.33	CI −0.02-0.61
Dotan et al. [7]	2014	Retrospective	25	0.312	0.043

### Mean visual loss following first versus second NAION events

**Fig. 4:**
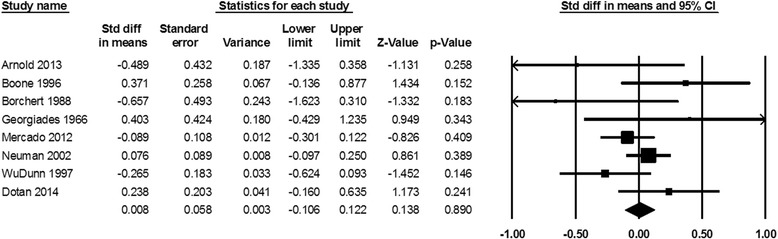
Standardized difference in means of visual acuity following the first versus the second NAION events

**Fig. 5:**
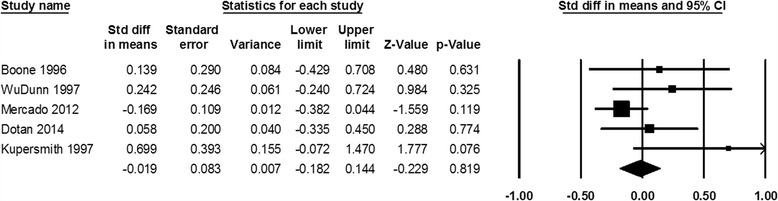
Standardized difference in means of visual field loss following the first versus the second NAION events

Mean visual acuity and visual field loss following the first NAION event are not significantly different compared with those after the second one (standardized mean difference of visual acuity 0.008, *P* = 0.890, and visual field loss −0.019, *P* = 0.819, Figs. 4, 5).

## Discussion

In this study, we conducted a systematic literature review and meta-analysis of visual outcome following bilateral NAION. Our analysis reached similar results for both visual acuity and visual field loss, finding a weak connection in vision in both eyes of the same patient. In addition, it was determined that there is no difference in severity of the first versus the second NAION events.

For patients suffering an event of NAION it is of great personal importance to know whether it is possible to predict the severity of consecutive event in the other eye, should it occur. The largest prospective study to date analyzing visual outcome following bilateral NAION is the “Ischemic Optic Neuropathy Decompression Trial Follow-up Study”, [3] reporting on 128 patients with bilateral NAION; 80 patients had prior NAION in the fellow eye before enrollment, and 48 patients experienced new NAION in their other eye during the course of the study. Bilateral NAION occurred both in patients randomized for optic nerve sheath decompression and in controls. In approximately half of the patients with bilateral NAION visual acuity in both eyes was within three lines of one another; however, in 30% of patients the visual acuity difference between the eyes was greater than 6 lines leading the authors to conclude that predicting visual outcome of the second eye based on initial presentation is problematic. In our meta-analysis we found significant correlations of visual acuity (*R* = 0.387) and visual field loss (*R* = 0.445) in both eyes of the same individual following bilateral NAION; however, they were relatively small, implying a weak similarity in these outcomes in both eyes. Based on these results the calculated coefficient of determination for visual acuity is 0.149 and for visual field loss it is 0.198, indicating that for any given individual less than 15% of visual acuity and 20% of visual field loss in the secondly affected eye can be explained by these outcomes in the firstly affected eye.

These findings support the findings of one of the authors of this meta-analysis (GD) who previously reported that that there is a stronger correlation for visual field loss than for visual acuity following bilateral NAION. In addition, it was reported that the amount retinal nerve fiber layer (RNFL) thinning measured by optical coherence tomography (OCT) is similar in both eyes. [7]. Since no other studies evaluated RNFL thickness similarity in both eyes following bilateral NAION this parameter was not included in this meta-analysis; and therefore, it is impossible for us to determine whether RNFL thinning is truly similar in both eyes.

Some authors reported that the second NAION event is usually milder as a result of better control of vasculopathic risk factors including initiation of anti-thrombotic therapy following the first event [8, 9]. On the other hand, others reported similar severity of both NAION events, [10] and the result of this meta-analysis support these studies.

A major strength of this study is that a comprehensive literature search identified all relevant reports, and the methodological quality of included studies was assessed using objective measures. Its limitations include the relative paucity of literature reports regarding bilateral NAION, and the nature of most studies which were retrospective case series with their inherent limitations of selection bias and availability of accurate and complete data recordings.

## Conclusions

Although a connection exists between visual outcome in both eyes following bilateral NAION the association is small, indicating that it is still impossible to predict with certainty the severity of a successive NAION event based on initial presentation. Patients suffering extensive visual loss following the first event can be advised that the outcome of a subsequent event, should it occur, may be much more favorable. In addition, there is currently insufficient evidence to support a difference in severity of the first versus the second NAION event.

## Abbreviations

LogMAR: Logarithm of Minimal Angle of Resolution
NAION: Non-Arteritic Anterior Ischemic Optic Neuropathy
OCT: Optical Coherence Tomography
RNFL: Retinal Nerve Fiber Layer
